# Birth-weight, insulin levels, and HOMA-IR in newborns at term

**DOI:** 10.1186/1471-2431-12-94

**Published:** 2012-07-07

**Authors:** Luis E Simental-Mendía, Argelia Castañeda-Chacón, Martha Rodríguez-Morán, Fernando Guerrero-Romero

**Affiliations:** 1 Biomedical Research Unit, Mexican Social Security Institute, Prédio Canoas 100, Col. Los Angeles, Durango, Dgo, 34067, Mexico

## Abstract

**Background:**

Recent studies have demonstrated that low and high birth-weight at birth are risk factors of developing diabetes. The aim of this study was to determine if the abnormal birth-weight is related with hyperinsulinemia and elevated index of the Homeostasis Model assessment for Insulin Resistance (HOMA-IR) at birth, in at term newborns.

**Methods:**

Newborns with gestational age between 38 and 41 weeks, products of normal pregnancies of healthy mothers aged 18 to 39 years, were eligible to participate. Small-for-gestational age (SGA) and large-for-gestational age (LGA) newborns were compared with appropriate-for-gestational (AGA) age newborns. Incomplete or unclear data about mother’s health status, diabetes, gestational diabetes, history of gestational diabetes, hypertension, pre-eclampsia, eclampsia, and other conditions that affect glucose metabolism were exclusion criteria. Hyperinsulinemia was defined by serum insulin levels ≥13.0 μU/mL and IR by HOMA-IR ≥2.60. Multiple logistic regression analysis was used to determine the odds ratio (OR) that computes the association between birth-weight (independent variable) with hyperinsulinemia and HOMA-IR index (dependent variables).

**Results:**

A total of 107 newborns were enrolled; 13, 22, and 72 with SGA, LGA, and AGA, respectively. Hyperinsulinemia was identified in 2 (15.4%), 6 (27.3%), and 5 (6.9%) with SGA, LGA, and AGA (*p*=0.03), whereas IR in 3 (23.1%), 8 (36.4%), and 10 (13.9%) newborns with SGA, LGA and AGA (*p*=0.06). The LGA showed a strong association with hyperinsulinemia (OR 5.02; CI 95%, 1.15-22.3; p=0.01) and HOMA-IR (OR 3.54; CI 95%, 1.03-12.16; p=0.02); although without statistical significance, the SGA showed a tendency of association with hyperinsulinemia (OR 2.43; CI 95%, 0.43-17.3 p=0.29) and HOMA-IR (OR 1.86; CI 95%, 0.33-9.37; p=0.41).

**Conclusions:**

Our results suggest that LGA is associated with hyperinsulinemia and elevated HOMA-IR at birth whereas the SGA show a tendency of association.

## Background

The type 2 diabetes is a complex disease characterized by decrease of insulin sensitivity and impaired insulin secretion [1]. Recent studies have demonstrated that low birth-weight is a risk factor for development of obesity and type 2 diabetes in adulthood [2], association that could be explained because during intrauterine growth the fetus does not promote the appropriate growth of beta cells [3,4]. Furthermore, children with history of high birth-weight also have an elevated risk of developing obesity and type 2 diabetes later in life [5-7]. The causal relationship between birth-weight and the presence of glucose metabolic disorders in the adulthood describe a U curve with the low and high birth-weight showing increased risk.

In addition to genetic predisposition, nutritional, and environmental factors, it has been reported that the elevated levels of cytokines increase glucocorticoid activity and decrease serum adiponectin during pregnancy, which affects birth-weight [8-11]. Furthermore, insulin, which is regulated by fetal tissue, is an essential endocrine regulator of intrauterine growth [12-14].

It has been described that in large-for-gestational age (LGA) infants, cord serum levels of leptin are strongly related with birth-weight and insulin levels, suggesting that part of insulin’s effect on birth-weight is mediated by leptin [15]. Furthermore, also it has been reported that conditions that modify insulin concentration during fetal life could alter the normal development of endocrine system, predisposing to insulin resistance in adult life [16,17].

Given that studies about association between birth-weight and insulin resistance has been developed measuring insulin and glucose levels in adolescence or adulthood and collecting information of birth-weight in a retrospective way, the aim of this study was to determine if the abnormal birth-weight is related with elevated insulin levels and increase of the Homeostasis Model assessment for Insulin Resistance (HOMA-IR) index at birth, in at term newborns.

## Methods

With protocol approval by the Mexican Social Security Institute Research Committee and after obtain the mother’s informed consent, a cross-sectional study of two phases was carried out.

In the first phase, with the purpose of establish the cutoff point of HOMA-IR at birth in appropriate-for-gestational age (AGA) newborns delivered at term, a total of 150 newborns with birth-weight greater than 2500 g and lower that 4000 g, were enrolled in a cross-sectional non-comparative study.

In the second phase, an independent sample of 107 newborns were enrolled in a cross-sectional comparative study to determine if LGA or small-for-gestational age (SGA) is related with hyperinsulinemia and elevated HOMA-IR in newborns delivered at term.

In both phases, eligible participants were newborns of mother’s aged 18 to 39 years and gestational age (determined by ultrasound) between 38 and 41 weeks. All newborns were of pregnant women without diagnosis of diabetes or gestational diabetes (confirmed by oral glucose tolerance test), history of gestational diabetes, high blood pressure, pre-eclampsia, eclampsia, asthma, epilepsy, or malnutrition.

In addition, in both phases the presence of congenital malformations, incomplete or unclear data about mother’s health status as well as preterm delivery, intrauterine infection, smoking or alcohol consumption during pregnancy, and the use of dextrose solutions during labor, or drugs that affect the glucose metabolism, were exclusion criteria.

In the second phase of the study, in order to control potential confounders related with maternal characteristics, the groups were matched by mother’s age, parity, and gestational age.

Anthropometric characteristics and blood samples were obtained by pediatricians at delivery room, immediately after delivery. Blood samples were collected from the umbilical cord vein.

Newborns were distributed in the groups in study according the presence of SGA, AGA, or LGA.

Detailed information regards mother’s health was collected by direct medical history, physical examination, and by reviewing their medical records of prenatal control.

The body mass index (BMI) of mothers before pregnancy was calculated as the weight (kilograms) divided by the height (meters) squared. The height and weight were assessed with a stadiometer using a fixed scale. The increments of the weight and height measurements were 0.1 kg and 0.01 m, respectively.

### Definitions

SGA, AGA, and LGA were defined by weight at birth <2500g, ≥2500<4000g, and >4,000g, respectively [18,19].

The HOMA-IR index was calculated using the formula: fasting insulin (μU/mL) x fasting glucose (mmol/L)/22.5 [20].

### Assays

Serum glucose was measured using the glucose-oxidase method (Sigma Diagnostics, St Louis, Mo, USA). The intra- and inter-assay coefficients of variation for glucose measurements were 2.1 and 2.8%. Insulin levels were measured by microparticle enzyme immunoassay (Abbott Axsym System, Alameda, CA, USA), with intra- and inter-assay variation coefficients of 4.1% and 6.2%.

### Statistical analysis

Differences between the groups were estimated using one-way ANOVA with post hoc Bonferroni test. The Log*n* was calculated to normalize skewed data.

Multiple regression logistic analysis adjusted by maternal weight before pregnancy and sex of newborn was used to determine the odds ratio (OR) that computes the association between birth-weight (independent variable) with hyperinsulinemia and HOMA-IR index (dependent variables).

The 95% confidence intervals (CI 95%) were determined, and a *P* value <0.05 defined statistical significance. Data were analyzed using the statistical package SPSS 15.0 (SPSS Inc., Chicago IL, USA).

## Results

The characteristics of the mothers and newborns enrolled in the first phase of the study are shown in Table 1.

**Table 1:** Characteristics of pregnant women and birth-weight of newborns appropriate-for-gestational age delivered at term, N = 150

Family history of diabetes, n (%)	58 (38.6)
Parity	2.4 ± 3.2
Mother’s age, years	25.2 ± 5.2
Weight before pregnancy, kg	60.2 ± 11.8
Body mass index before pregnancy, k/m^2^	23.8 ± 4.3
Weight gain through pregnancy, kg	12.6 ± 4.0
Gestational age, weeks	39.2 ± 1.2
Newborn’s birth weight, g	3278 ± 321

Distributions of insulin, glucose, and HOMA-IR at birth of AGA newborns are shown in Table 2; the 90^th^ percentile of distribution for insulin levels was 13.0 μU/mL and the fourth quartile of HOMA-IR index 2.60. In the absence of cutoff points for insulin levels and HOMA-IR index in the newborn at term, these values were considered as the cutoff points for the second phase of the study.

**Table 2:** Characteristics of the target population according to birth weight, n = 107

	Overall population	SGA	AGA	LGA		
	n = 107	n = 13	n = 72	n = 22	F	*p*
Family history of diabetes, n (%)	45 (40.1)	8 (61.5)	26 (36.1)	11 (50.0)	-	0.13
Parity	2.2 ± 1.0	2.3 ± 1.2	2.0 ± 1.0	2.4 ± 1.0	0.94	0.39
Mother’s age, years	25.0 ± 5.0	23.0 ± 6.1	24.8 ± 5.2	26.6 ± 3.9	2.14	0.12
Weight before pregnancy, kg	61.8 ± 11.7	55.9 ± 11.8	61.1 ± 10.6	67.5 ± 13.2	4.60	0.01^*, **,†^
Weight gain through pregnancy, kg	13.0 ± 5.2	11.1 ± 6.7	12.8 ± 5.3	14.7 ± 3.2	1.95	0.14
Body mass index before pregnancy, k/m^2^	24.1 ± 4.6	22.1 ± 5.1	23.9 ± 4.0	25.8 ± 5.5	2.8	0.06
Gestational age, weeks	39.1 ± 1.8	39.0 ± 0.8	39.3 ± 1.2	39.8 ± 0.9	2.22	0.11
Newborn’s birth weight, g	3312 ± 678	2083 ± 356	3270 ± 380	4175 ± 197	149.3	<0.001^*,**,†,‡,§^
Newborn’s plasma glucose at birth, mmol/L	5.9 ± 2.7	7.2 ± 5.5	5.8 ± 2.0	5.2 ± 2.2	2.15	0.15
Newborn’s insulin at birth, μU/mL	7.1 ± 4.3	6.7 ± 4.5	6.3 ± 3.7	9.6 ± 4.9	5.32	0.001^‡^
HOMA-IR index	1.9 ± 1.5	1.9 ± 1.4	1.7 ± 1.3	2.3 ± 1.5	1.63	0.04^‡^

Values are median ± standard deviation.

SGA, small-gor-gestational age; AGA, appropriate-for-gestational age; LGA, large-for-gestational age.

*p < 0.01 between SGA and LGA. **p < 0.005 between SGA and the overall population. ^†^p < 0.005 between LGA and the overall population.

^‡^p < 0.05 between LGA and AGA. ^§^p = 0.04 between SGA and AGA.

In the second phase of the study, 13 (12.1%), 22 (20.6%), and 72 (67.3%) newborns delivered at term were enrolled in the SGA, LGA, and AGA groups, respectively.

Clinical and biochemical characteristics of the mothers and newborns in the second phase of the study are shown in Table 3.

**Table 3:** Distribution of cord serum levels of insulin, glucose and HOMA-IR index in newborns appropriate-for-gestational age delivered at term, N = 150

		Percentiles				
	Mean ± SD	5	25	50	75	90
Newborn’s insulin at birth, μU/mL	8.2 ± 8.8	2.42	3.90	5.60	8.80	13.0
Newborn’s plasma glucose at birth, mmol/L	6.1 ± 1.9	3.9	4.7	5.6	6.2	6.7
HOMA-IR	2.2 ± 2.5	0.52	0.94	1.51	2.60	2.89

The mother’s weight and BMI before pregnancy was significantly lower in the mothers of SGA newborns as compared with mother’s of LGA and AGA newborns. The insulin levels in the newborns were significantly higher in the LGA group as compared with the SGA and AGA groups. Other variables showed no significant differences between the groups in study.

Hyperinsulinemia was identified in 13 (12.1%) newborns: 2 (15.4%), 5 (6.9%), and 6 (27.3%) in the groups with SGA, AGA, and LGA (*p*=0.03).

Elevated HOMA-IR was detected in 21 (19.6%) newborns: 3 (23.1%), 10 (13.9%), and 8 (36.4%) in the groups SGA, AGA, and LGA (*p*=0.063).

The LGA show a strong association with hyperinsulinemia (OR 5.02; CI 95%, 1.15-22.3; p=0.01) and HOMA-IR (OR 3.54; CI 95%, 1.03-12.16; p=0.02); the SGA showed a tendency of association with hyperinsulinemia (OR 2.43; CI 95%, 0.43-17.3 p=0.29) and HOMA-IR (OR 1.86; CI 95%, 0.33-9.37; p=0.41).

## Discussion

Results of this study suggest that at LGA newborns have higher insulin levels and HOMA-IR than AGA and SGA newborns.

It has been suggested that the association between LGA and insulin resistance may reflect the influence of several factors like maternal diabetes and gestational age [21-24]; in the second phase of the study, to control these potential confounders, the mother’s age, parity, and gestational age of mothers in the groups of newborns with SGA, AGA, and LGA, were matching criteria.

Our results agree with the report by Wolf et al. [15] who found in the cord blood of 175 neonates with a birth-weight of 3376±588g, insulin levels of 7.4±4.3 μU/mL as compared with our results in 107 at term newborns with birth-weight and insulin levels of 3313±678g and 7.1±4.3 μU/mL. However, the increase of insulin levels, according birth-weight class, in the study by Wolf et al. (SGA<AGA<LGA) showed a different sequence as compared with our results (AGA<SGA<LGA); finding that could be related with differences in the birth-weight of the LGA newborns, which were heavier (3824±364g versus 4175±197g), and/or the birth-weight of SGA newborns (2516±352g versus 2083±356g) that were lighter in our study as compared with the report by Wolf et al. [15]. Finally, in addition to leptin, also differences in glucose levels could explain differences in the sequence of insulin concentration; given that we did not measure leptin and Wolf et al. [15] did not report glucose levels, the results are not comparable in this issue. However, because criteria to define SGA and LGA at birth were not the same, the comparison between our results and those from Wolf et al. should be taken cautiously.

Furthermore, in our study the sequence by weight class in the newborn at birth, for glucose and HOMA-IR was LGA<AGA<SGA and AGA<LGA<SGA, respectively, suggesting that LGA newborns at birth exhibited a decrease of insulin sensitivity, that requires an elevated compensatory insulin secretion from fetus to maintain euglycemia.

Recently, Catalano et al. [25] conducted a comparative study that included 53 lean and 68 obese pregnant women to verify if fetuses of obese women have increased insulin resistance. Although we did not analyze our data based on the BMI of pregnant women, our results agree with the report by Catalano et al. On this regard, the birth-weight of newborns of lean and obese women (3217±452g and 3320±460g) in the study by Catalano et al. [25] is similar to the birth-weight of AGA newborns in our study (3270±380g), as was the HOMA-IR (1.51±0.86 vs. 1.06±0.70 in the newborns of obese and lean mothers, in the study by Catalano) and 1.7±1.3 in the AGA newborns of our study. Furthermore, Catalano et. al. [25] also reported that there was a positive correlation between fetal insulin resistance and fetal adiposity supporting our finding that suggest that elevated body-weight at birth might be the main risk factor involved in the development of hyperinsulinemia and elevated HOMA-IR in the newborns.

Interestingly, although SGA and AGA newborns exhibited similar HOMA-IR, blood cord glucose levels were higher in the SGA newborns, suggesting that could be an impaired insulin secretion and or insulin action related with the low birth-weight at birth. Additional factors related with the insulin production by fetus, such as elevated leptin and decreased adiponectin levels also could explain the findings of our study in regards SGA newborns. Further studies with the appropriate biochemical measurements are needed.

Recently, in the Hyperglycemia and Adverse Pregnancy Outcome Study, it has been reported that cord serum C-peptide levels are strongly related with percent body fat of newborns and mothers glycemia [26]; although we have not data about mothers glycemia, our results based in measurement of cord insulin levels immediately after delivery, are in agree with these finding and support the statement of a strong relationship between increase of insulin by fetus and the increase of body weight at birth.

In regards the association between birth-weight and insulin resistance later in life, children and young adults with history of very low birth-weight and preterm delivery exhibit higher indices of HOMA-IR as compared with newborns delivered at term [27-29]; given that SGA newborns have accelerated weight and fat mass gain during first years of life is expected the development of insulin resistance later in life in these children [30]. Taking into account that SGA newborns in our study exhibited low HOMA-IR, is probable that the increase of leptin levels related with the increase of fat tissue have an important role in the transition from low insulin secretion and HOMA-IR at birth to high insulin secretion and HOMA-IR at childhood. Further research is needed in the field to clarify underwent mechanisms involved in the fetal insulin secretion.

An additional contribution of our study was the reference values of cord insulin levels and HOMA-IR in AGA newborns. On this regard, because there are not reference values of insulin and HOMA-IR at birth, we evaluate its distribution in an independent group of AGA newborns who had the same inclusion and exclusion criteria that newborns enrolled in the second phase of this study. In this way, we obtained the percentile distribution of insulin levels and HOMA-IR as reference value of both variables.

Finally, it is necessary to keep in mind that mechanisms involved in the relationship between fetal growth and insulin resistance have not been established with certainty; therefore, further studies in the field are necessaries.

Several limitations deserve to be mentioned. First, the small sample size of LGA or SGA newborns in this study could be a source of bias that affects our conclusion. However, given the inclusion and exclusion criteria of this study, is unlikely to find newborns at term with LGA or SGA; further research that includes multiple centers is necessary to increase the sample size in order to confirm our results. Second, the cause-effect relationship cannot be established with certainty in the cross-sectional design studies; however, other methodological designs could involve ethical issues.

The homogeneous target population and the strict matching criteria for controlling the main confounders were the main strengths of this study. In addition, given that pregnant women were in prenatal control, we have reliable data about maternal characteristics.

## Conclusion

In conclusion, our results suggest that LGA is associated with hyperinsulinemia and elevated HOMA-IR at birth whereas SGA show a tendency of association.

## Competing interests

The authors have not conflict of interest and nothing to disclose.

## Authors’ contributions

LE. S-M, AC-C, MR-M, and FG-R had full access to all of the data in the study and take responsibility for the integrity of the data and the accuracy of the data analyses. Study concept and design: LE. S-M, FG-R, MR-M. Acquisition of data: AC-C, MR-M. Analysis and interpretation of data: LE S-M and FG-R. Drafting of the manuscript: LE. S-M. Critical revision of the manuscript for important intellectual content: MR-M, and FG-R. Statistical analysis: LE. S-M. Obtained funding: MR-M and FG-R. Administrative, technical, and material support: AC-C. Study supervision: MR-M. All authors read and approved the final manuscript.

## Pre-publication history

The pre-publication history for this paper can be accessed here:

http://www.biomedcentral.com/1471-2431/12/94/prepub
